# SARS-CoV-2 alters neural synchronies in the brain with more severe effects in younger individuals

**DOI:** 10.1038/s41598-023-29856-7

**Published:** 2023-02-20

**Authors:** Helen Valsamis, Samah Abdul Baki, Jason Leung, Samer Ghosn, Brittany Lapin, Geetha Chari, Izad-Yar Rasheed, Jaehan Park, Vineet Punia, Ghinwa Masri, Dileep Nair, Ann Marie Kaniecki, Muhammad Edhi, Carl Y. Saab

**Affiliations:** 1 Kings County Hospital, Brooklyn, NY USA; 2 SUNY Health Sciences University, Brooklyn, NY USA; 3 Bio-Signal Group Corp, Acton, MA USA; 4 Cleveland Clinic Foundation, Cleveland, OH USA; 5 American University of Sharjah, Sharjah, UAE; 6 Case Western Reserve University, Cleveland, OH USA; 7 Brown University, Providence, RI USA

**Keywords:** Computational biology and bioinformatics, Neuroscience, Neurology

## Abstract

Coronavirus disease secondary to infection by SARS-CoV-2 (COVID19 or C19) causes respiratory illness, as well as severe neurological symptoms that have not been fully characterized. In a previous study, we developed a computational pipeline for the automated, rapid, high-throughput and objective analysis of electroencephalography (EEG) rhythms. In this retrospective study, we used this pipeline to define the quantitative EEG changes in patients with a PCR-positive diagnosis of C19 (n = 31) in the intensive care unit (ICU) of Cleveland Clinic, compared to a group of age-matched PCR-negative (n = 38) control patients in the same ICU setting. Qualitative assessment of EEG by two independent teams of electroencephalographers confirmed prior reports with regards to the high prevalence of diffuse encephalopathy in C19 patients, although the diagnosis of encephalopathy was inconsistent between teams. Quantitative analysis of EEG showed distinct slowing of brain rhythms in C19 patients compared to control (enhanced delta power and attenuated alpha–beta power). Surprisingly, these C19-related changes in EEG power were more prominent in patients below age 70. Moreover, machine learning algorithms showed consistently higher accuracy in the binary classification of patients as C19 versus control using EEG power for subjects below age 70 compared to older ones, providing further evidence for the more severe impact of SARS-CoV-2 on brain rhythms in younger individuals irrespective of PCR diagnosis or symptomatology, and raising concerns over potential long-term effects of C19 on brain physiology in the adult population and the utility of EEG monitoring in C19 patients.

## Introduction

Coronavirus disease (COVID19, abbreviated here as C19) is caused by infection with the SARS-CoV-2 virus. Most people infected with the virus will experience mild to moderate respiratory illness, and individuals with underlying medical conditions, especially older people, appear to be more vulnerable (World Health Organization, https://www.who.int/health-topics/coronavirus#tab=tab_1). Irrespective of these risk factors, and for incompletely understood reasons, some will become seriously ill and manifest severe neurological symptoms requiring admission to an intensive care unit (ICU). Although evidence related to the presence of SARS-CoV-2 in the central nervous system (CNS) is sparse, and direct viral invasion of the CNS is difficult to estimate^1,2^, the significant impact of the virus on the CNS and its contribution to neurological sequelae are uncontested, partly as a result of immune-mediated or autoimmune reactions leading to neuro-inflammation^3^.

Neurological symptoms of C19 include loss of smell and seizures^4,5^, and more broadly defined symptoms such as brain ‘fogginess’, dizziness, extreme fatigue, and sleepiness^6,7^. In cases where electroencephalography (EEG) has been performed on C19 patients, predominant observations converge on generalized slowing and diffuse encephalopathy^8–19^. Invariably, however, these observations have been based on visual interpretation of the EEG, a method that is known to be qualitative and time consuming. Conventional EEG systems require significant training and expertise for accurate visual interpretation of EEG, in particular for the removal of artifacts which exacerbate inter-rater variability and sampling bias^20–22^. These concerns are more salient when dealing with C19 patients, especially in the ICU. Even though subjective interpretation of EEG led many studies to conclude that EEG patterns are significantly altered in C19 patients^8–19^, primarily in frontal lobes^8,12,19^ and within distinct low frequency bands of delta^12^ and alpha^15^, detailed, quantitative and objective characterization of these patterns has only been achieved in two studies with low sample sizes^14,23^. A few studies have even suggested that EEG is visually normal with scarce abnormalities^24^ or indistinguishable from other pathological conditions^25^. Moreover, widely held assumptions about milder neurologic symptoms in the pediatric population have been challenged^26,27^. Lack of adequate control groups in several EEG studies is a major concern^14^, especially with regards to age which is a significant variable in EEG research^28,29^. Hence, there is an urgent unmet need for the standardization of quantitative EEG analysis for the discovery of objective, physiological markers that could help in the diagnosis, prognosis and monitoring of therapy in critically ill patients with C19^14,23,24^.

Rhythmic brain activity or ‘oscillation’ in discrete frequency bands, a fundamental characteristic of spontaneous activity in mammalian brain covering large scale neural networks, is a hallmark of mental states in health^30–32^ and disease^33^. The study of spontaneous brain oscillations reveals subtle, emergent properties of dynamic and high-speed brain function related to mental states in real-time. Therefore, we postulated that the analysis of EEG oscillations in C19 patients with CNS symptoms could provide proof of concept data to guide the discovery of future novel markers of the disease. A comprehensive and detailed assessment of oscillations, including power amplitude in distinct frequency bands and temporal coupling between bands, cannot be achieved reliably by visual inspection alone. Therefore, our laboratory has developed a computational pipeline for the automated, rapid, high-throughput and objective analysis of EEG oscillations. This pipeline is based on a support vector machine (SVM) algorithm for the accurate detection of EEG artifacts, which has been validated in rodent, non-human primate and human subjects^34^.

In this retrospective study including 69 subjects, we investigated the quantitative changes in EEG oscillations of patients with a PCR-positive diagnosis of C19 (n = 31) in the ICU at Cleveland Clinic, compared to a group of PCR-negative (n = 38) patients in the same ICU. Clinical reviews of EEG by visual inspection conducted by clinical fellowship trainees and board certified electroencephalographers at two independent sites (Cleveland Clinic, OH and King’s County/SUNY, NY) confirmed prior reports with regards to the high prevalence of diffuse encephalopathy in C19 patients, albeit to the same degree observed in age-matched control patients. Moreover, diagnoses of encephalopathy at the individual subject level were not fully consistent across both sites. Quantitative EEG analysis, however, showed distinct slowing of EEG in C19 subjects compared to control subjects, which was more severe in subjects below age 70, such that power distribution in younger subjects in the low frequency bands (< 35 Hz) was indistinguishable from that in subjects above age 70. These results were further corroborated using an unbiased approach based on machine learning, whereby the accuracy of several algorithms for the binary classification of patients as C19 versus control using EEG power features was consistently higher for patients below age 70, raising concern about the long-term effects of C19 in younger age population and the utility of EEG monitoring in C19 patients of all ages.

## Results

The mean length of hospitalization prior to the EEG recording was not statistically significant between C19 positive patients (4.7 ± 1.3 days) and C19 negative patients (3.1 ± 1.2, *p* = 0.34). All channels rejected in our study were based on qualitative inspection, and none were rejected based on automated identification of artifacts. In the Ct group, 2 EEG recordings contained 2 rejected channels, and 1 recording contained 5 rejected channels; in the C19 group, 1 EEG recording contained 2 rejected channels. Mean artifact-free EEG epoch durations per subject in C19 and Ct groups were 663 ± 20 s and 698 ± 23 s, respectively. Neuropsychiatric comorbidities were identified in both groups but with low prevalence (n = 6 out of 38 in C19 group and n = 3 out of 31 in Ct group).

**Figure 1:**
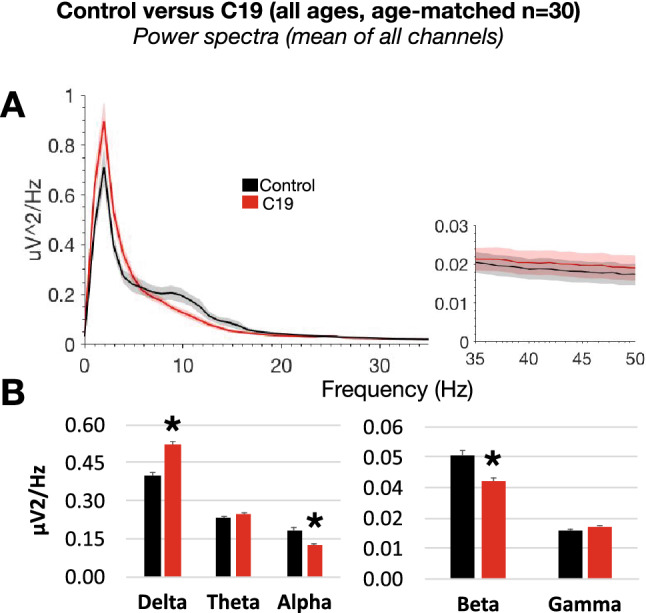
(**A**) Power spectral density (mean of 16 EEG channels) in age-matched control and C19 subjects (n = 30 per group) in the 0–50 Hz frequency range. (**B**) Mean power in the frequency bands delta (1–4 Hz), theta (5–9 Hz), alpha (10–13 Hz), beta (14–32 Hz) and low gamma (33–52 Hz).

**Figure 2:**
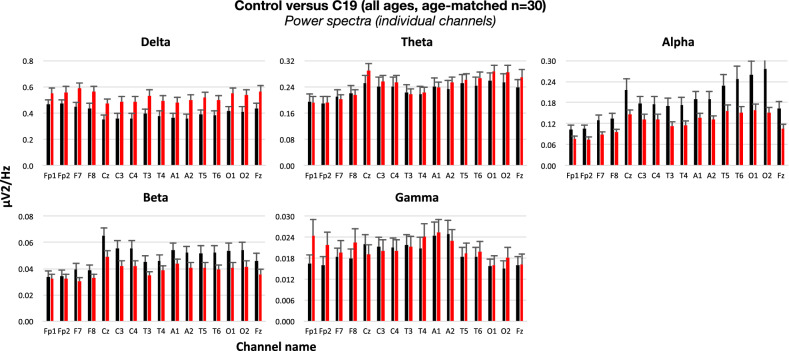
Power spectral density in 16 individual EEG channels in age-matched control and C19 subjects (n = 30 per group) in the frequency bands delta (1–4 Hz), theta (5–9 Hz), alpha (10–13 Hz), beta (14–32 Hz) and low gamma (33–52 Hz). No statistically significant difference was noted between groups in any individual channel.

Visual interpretation of EEG by two independent teams of trained electroencephalographers at Cleveland Clinic and SUNY resulted in normal or encephalopathy diagnosis using standard clinical criteria including diffuse delta slowing (Table 1). Overall, this qualitative diagnosis was inconsistent among both teams, and more so for the C19 group (14 inconsistent diagnoses out of 31 or 45%) versus control (7 inconsistent diagnoses out of 38 or 22%). When subjects were age-matched (Sup Fig. 1), quantitative analysis of the mean EEG power showed distinct and significant differences in C19 compared to the control group (Fig. 1), including an increase in delta (0.52 ± 0.01 in C19, 0.40 ± 0.01 in Ct, *p* < 0.001), and a decrease in alpha (0.12 ± 0.007 in C19, 0.18 ± 0.01 in Ct, *p* < 0.01) and beta power (0.038 ± 0.002 in C19, 0.048 ± 0.002 in Ct, *p* < 0.01). Analysis of power in individual 16 channels further showed that power changes were not localized to particular brain areas (Fig. 2).

**Table 1:**
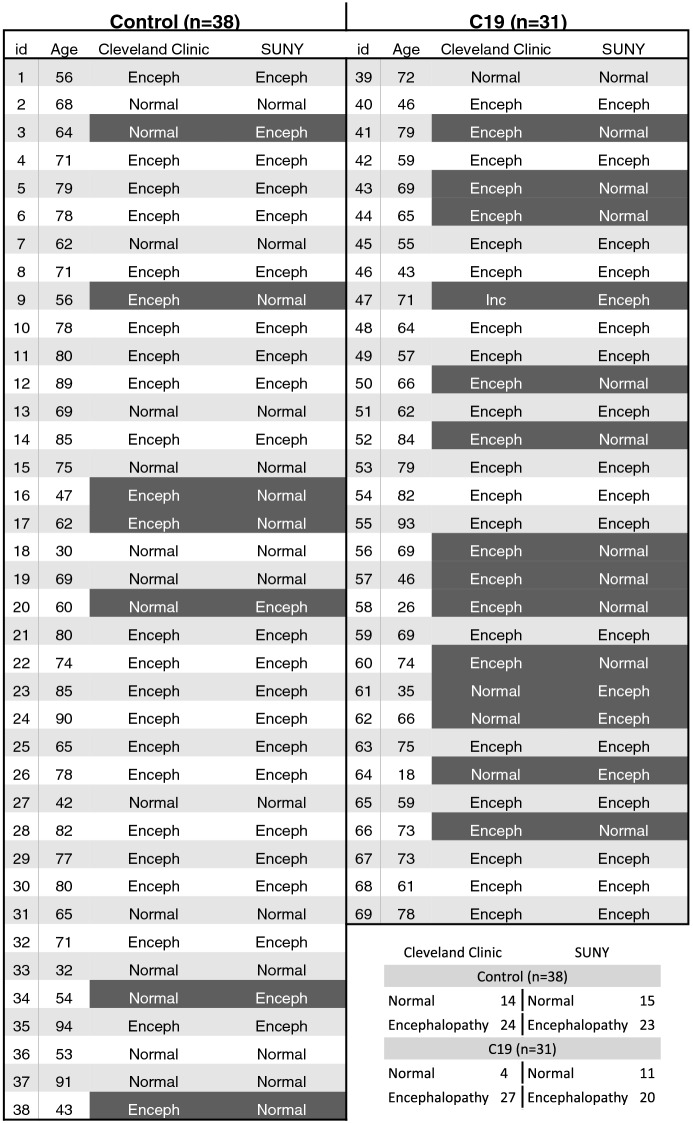
Qualitative assessment of EEG by two independent teams at Cleveland Clinic and SUNY for the diagnosis of encephalopathy (n = 38 control, n = 31 C19).

**Figure 3:**
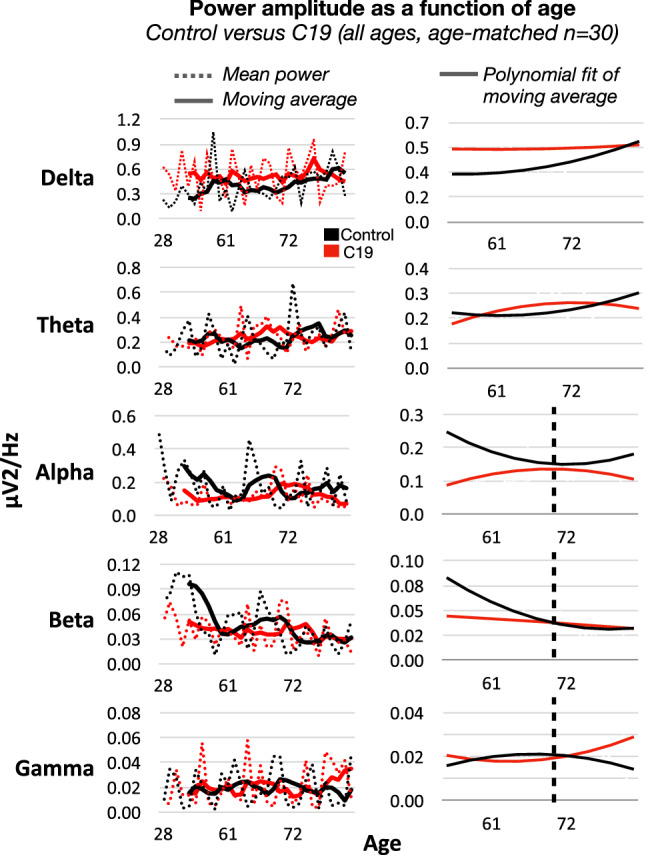
(Left panel) Power values (mean of 16 channels) were plotted as a function of age in control and C19 subjects, superimposed over 3-steps moving averages in each group, for delta, theta, alpha, beta and low gamma bands. Of note, X-axis values represent the average age for each pair of subjects based on the matching requirements of maximum 5 years difference (i.e. X-axis is non-linear). (Right panel) Moving averages shown in left panel were fitted with polynomial trend lines, which were convergent or intersecting around age 70 for alpha, beta and gamma (vertical lines). This formed the basis of group selection by age below and above 70 years.

Furthermore, when power within individual frequency bands was plotted against age, and moving averages of these plots were fit to polynomial trend lines, a crossover in power amplitude was noted around age 70 (Fig. 3). Noting that this crossover timeline was approximate (because it was generated based on matching requirements within 5 years of age), age 70 was then used to divide subjects within a group as younger (i.e. below 70 age) or older groups (i.e. above 70 age), and to subsequently test the effect of age on power changes in C19 patients compared to control.

**Figure 4:**
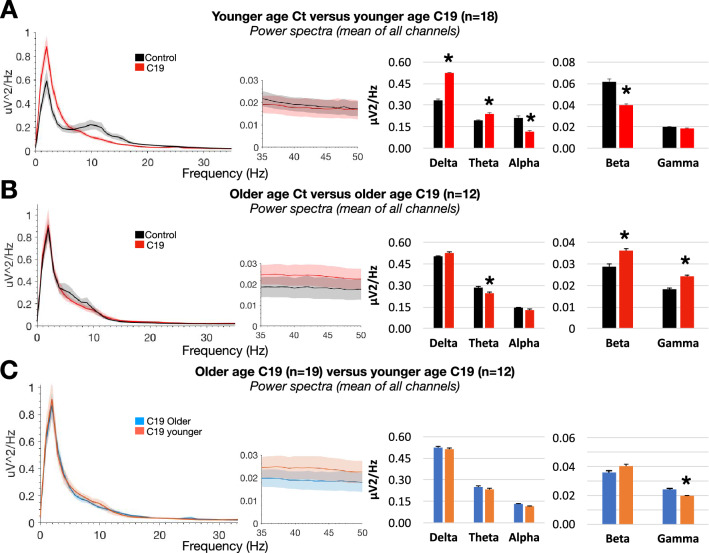
(**A**) Power spectral density (mean of 16 EEG channels) in age below 70 (younger) control and age below 70 (younger) C19 subjects (n = 18 per group). Histograms show power in individual frequency bands. (**B**) Same as (**A**) for age above 70 (older) control and age above 70 (older) C19 subjects (n = 12 per group). (**C**) Same as (**A**–**B**) for age above 70 (older) C19 subjects (n = 19) and age below 70 (younger) C19 subjects (n = 12).

Results indicate that the power changes observed between age-matched C19 and control groups is further accentuated when the analysis is restricted to only younger subjects (Fig. 4), whereby delta and theta are significantly increased in the C19 group compared to control (0.522 ± 0.009 in C19, 0.334 ± 0.012 in Ct, *p* < 0.001 for delta, and 0.241 ± 0.007 in C19, 0.195 ± 0.003 in Ct, *p* < 0.001 for theta), whereas alpha and beta are significantly decreased (0.118 ± 0.006 in C19, 0.210 ± 0.015 in Ct, *p* < 0.001 for alpha, and 0.040 ± 0.001 in C19, 0.061 ± 0.002 in Ct, *p* < 0.001 for beta). Analysis of power within individual channels did not show localized changes (Sup Fig. 2), and phase-amplitude coupling between gamma and lower frequency bands was not changed (Sup Fig. 3). On the other hand, older C19 subjects showed a different pattern of power changes relative to younger subjects, such as decreased theta (0.247 ± 0.010 in C19, 0.286 ± 0.008 in Ct, *p* < 0.05) as well as increased beta and gamma (0.036 ± 0.001 in C19, 0.028 ± 0.001 in Ct, *p* < 0.05 for beta, and 0.024 ± 0.001 in C19, 0.018 ± 0.0007 in Ct, *p* < 0.001 for gamma). However, no statistically significant power change was observed at the single channel level (Sup Fig. 4). These data prompted a comparison within C19 group between younger versus older subjects, which showed no significant change in power except a decrease in gamma in younger subjects (0.019 ± 0.0006 in younger, 0.024 ± 0.001 in older, *p* < 0.01), results that were consistent with the lack of statistically significant power change at the single channel level (Sup Fig. 5). A summary of mean power changes in all channels is provided in Table 2. Lastly, statistical analysis of potentially confounding clinical variables between groups using exact logistic regression to predict C19 status from each parameter separately, with linear age added to each model, revealed no significant difference (Sup Fig. 6).

**Table 2:** Summary of per cent power changes in Figs. 1 and 4.

	Age-matched Ct versus C19		Younger Ct versus C19		Older Ct versus C19		Younger versus older C19	
	%	+ /−	%	**+ /−**	%	+ /−	%	+ /−
Delta	30	+	56	+	ns	O	ns	O
Theta	ns	O	23	+	− 13	−	ns	O
Alpha	− 33	−	− 43	−	ns	O	ns	O
Beta	− 20	−	− 34	−	25	+	ns	O
Gamma	ns	O	ns	O	30	+	− 19	—

Machine learning algorithms for the binary classification of subjects as C19 versus control based on EEG recording reached consistently higher accuracy levels when trained on datasets in the younger age population (Table 3), with a mean of 70.5% accuracy in younger subjects compared to a mean of 55.6% for older subjects. These values were obtained using EEG power features selected based on the results of statistical analysis obtained in these groups (for example mean delta, theta, alpha and beta for younger subjects, and theta, beta and gamma for older subjects). Moreover, when the same feature selection used to train a given algorithm in one group was applied to the other group for direct comparisons of accuracy, algorithms for the younger subjects still performed better than those in older ones (77.2% vs. 47.5, and 69.4% vs. 55.6%, respectively).

**Table 3:**
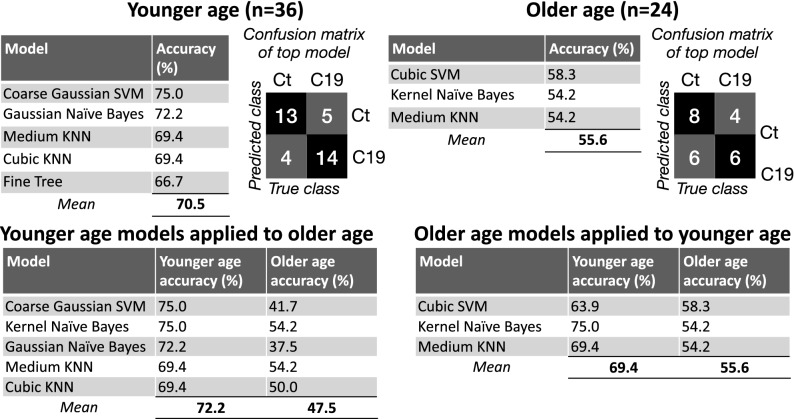
(Upper row) Accuracy results of machine learning algorithms trained on EEG power features selected based on statistical tests performed in Figs. 1 and 4 (for example in Fig. 1 mean delta, theta, alpha and beta for younger subjects, and theta, beta and gamma for older subjects).

(Bottom row) Feature sets used to train algorithms in a given group were applied to the other group for direct comparison. Only models with accuracy > 50% are shown.

## Discussion

The neurological symptoms of SARS-CoV-2 range from mild headache to altered mental status and seizures^4–7^. Assessment of these symptoms is based primarily on subjective criteria, self-reported questionnaires, and EEG when clinically indicated. However, the scientific literature on EEG and C19 is almost exclusively based on visual interpretation of EEG waveforms. This is a major concern because artifacts are ubiquitous in EEG and contribute to inter-rater variability and sampling bias^20–22^. Moreover, EEG analysis only in the time domain, and disregard to age as a significant variable, are important factors that compromise rigor and confound the interpretation of prior studies^14^. Therefore, we implemented an automated analytical pipeline for the analysis of EEG in the frequency domain to investigate neural oscillations in the brains of patients with C19, and compared those to control subjects in the same clinical environment but with a negative C19 diagnosis.

Our visual, qualitative analysis of EEG suggests that diffuse encephalopathy was highly prevalent in C19 patients, as well as in control patients. However, diagnosis was inconsistent between two independent sites at the individual patient level. Although this finding confirms prior reports about the high incidence of C19-related encephalopathy^8–19^, it highlights the challenges of EEG assessment based on visual inspection.

Our objective, quantitative analysis of the EEG data showed that the average power spectrum across EEG channels in C19 patients is significantly enhanced in the delta band, and attenuated in the alpha and beta bands compared to aged-matched control patients in the ICU with a negative C19 PCR test. Corroborating the diffuse characteristics of the encephalopathy, changes within these distinct frequency bands appear to be non-localized to individual channels. Moreover, we report that phase-amplitude coupling within individual channels is not significantly changed in C19 patients compared to control, indicating that the EEG changes in power do not necessarily translate to a generalized disruption in cross-frequency coupling between fast (gamma) and slow (delta-to-beta) bands, a proxy for effective brain communication.

These results related to oscillations, obtained in a fully-automated approach that enhances rigor and reproducibility, corroborate prior observations using visual inspection of the EEG, although past studies did not provide consistent and numerical estimates for these changes, nor accurate localization to specific channels and brain areas. According to a systematic review and meta-analysis, the proportion of abnormal EEG in C19 subjects is as high as 96%^16^. Non-specific, generalized background or diffuse slowing has been reported in several studies as the most common finding^10,11,18^, whereby EEG abnormalities correlate with encephalopathy, disease severity^8,9,17^, and risk for novel seizures^13^, including psychogenic non-epileptic seizures^5^. In particular, delta waves with absence of epileptic activity have been documented^12^, as well as alpha coma patterns suggestive of SARS-CoV-2 neurotropism for the brainstem ascending reticular system^15^. Although some studies suggested that SARS-CoV-2 could preferentially and directly target the frontal lobes^19^, with EEG abnormalities localized to frontal electrodes^8,12^, our results did not confirm these qualitative observations. The mechanisms underlying these observations are largely unknown; however, it has been suggested that metabolic hypoxia^18^, immune-mediated neuro-inflammation^3^, as well as severe stress and sleep disturbances^5^, are likely contributing factors.

Further analysis revealed evidence for age-dependent variations in power, with a clear threshold for differential effects around 70 years. Analysis of power in the younger age group (below age 70) showed significantly enhanced power in the delta and theta bands, and attenuated power in the alpha and beta bands, compared to the older population (above age 70). These power changes in younger individuals were not localized to individual channels, but more prominent in magnitude when compared to all ages combined. In older subjects, the power patterns were different from those in younger subjects, with significantly attenuated power in theta, and enhanced power in beta and gamma. However, these changes within the older population were less severe compared to those in the younger population, and were not localized to individual channels. Lastly, when power distribution in younger age C19 patients was compared to older ones with C19, no change in power was noted in lower frequency bands below 35 Hz, although gamma was significantly higher in older patients (results summarized in Table 2). To date, no study has investigated the age-dependency of C19 on dynamic brain physiology. Our results highlight the fact that the changes in EEG patterns affect younger subjects more than older ones, which is surprising given the general dogma that older individuals are at higher risk of developing severe C19 symptoms. Vulnerability of pediatric patients with multi-system inflammatory syndrome has been previously documented^26^, whereby long-term EEG monitoring after healing is recommended^27^. Hence, our results emphasize the need for continuous, long-term EEG monitoring for C19 patients in the ICU regardless of age, whereby we have shown that EEG power distribution in the low frequency range of patients below age 70 is indistinguishable from that above age 70. They further raise critical questions about reversibility and the potential long-term effects of C19 in younger individuals. Interestingly, the pattern of results regarding EEG slowing bares some resemblance to individuals with cognitive impairment secondary to Alzheimer’s disease^35,36^. However, the prevalence of dementia and/or Alzheimer’s in our study is negligible.

The results of our data-driven and statistically-guided machine learning algorithms further demonstrate that the accuracy of binary classification is consistently higher for younger individuals. Machine learning is based on the recognition of patterns and their representation (in our case without prior knowledge of data attributes other than binary labeling attached to C19 positive versus C19 negative input categories). In general, the more clearly delineated patterns are in the features used to train an algorithm (for example separation in numerical values between labels), the higher the algorithm’s performance. This suggests that the EEG features in younger adults are more distinct, and changes are more pronounced in C19 positive versus C19 negative labels, whereas in older individuals these features seem to be less distinct between labels. This assumption is well supported by the statistical analysis of the quantitative EEG.

The limitations in this study include the retrospective design, which was constrained by the need to collect EEG data from critically ill patients with a highly infectious disease during a pandemic, and the inability to control important variables such as medication and underlying medical conditions, although it is noteworthy that the significant EEG slowing in this study withstood this variability. Though there may have been some differences between the C19 and the age-matched cohorts in terms of anesthetics, systemic dysfunctions, etc. the small sample size precludes adjusting for all of these variables in our analysis, noting that both cohorts had a fairly well-matched length of hospitalization and mental status despite these possible differences, which correlates with the depth of EEG slowing on naked eye clinical examination. Moreover, interaction between several clinical conditions and age were excluded. While non-neuropsychiatric medical conditions can affect the depth of EEG slowing, we are not aware of specific EEG findings (e.g. focal or generalized slowing) that correlate with various other medical comorbidities, for example depression or anxiety. Moreover, our conclusions might not generalize to C19 patients outside of an ICU setting with less severe neurological symptoms. These attenuating gaps will be addressed in future prospective studies, whereby our quantitate methodology suggests a valid approach for developing diagnostic, prognostic and therapy monitoring of CNS symptoms secondary to C19 using machine learning and quantitative EEG. EEG is a cost-effective and non-invasive technique which can be potentially used as a bed-side tool to correlate with and monitor cognitive abilities, or predict risk-factors and future C19 long-haul at initial period. In addition, our analytical pipeline may prove useful for the objective and rapid evaluation of EEG findings in individuals who sustain lasting cognitive changes as seen in “long Covid”.

## Methods

### Study population

The study was approved by the Cleveland Clinic Institutional Review Board and the SUNY Downstate Health Sciences University Institutional Review Board & Privacy Board, which waived the requirement for patient consent. All methods were performed in accordance with relevant guidelines and regulations. We cross-matched the Cleveland Clinic C19 registry with the Cleveland Clinic EEG database (Ebase, Cleveland, OH) from April 20th, 2020 until August 20th, 2020. The main indication for EEG was concern for non-convulsive seizure contributing to altered mental status, or motor events concerning for seizure. All hospitalized C19 adults (≥ 18 years of age at the time of diagnosis of C19) who underwent an EEG during wakefulness resting states were included in the study population, excluding patients in coma, stupor or intubation. Patients were not instructed to have eyes open or closed. Patients were excluded if they did not undergo EEG evaluations during their admission for C19 infection. We identified n = 69 ICU patients (n = 31 C19; n = 38 Control, age-matched to C19 within 5 years of age) who underwent a 20-min screening EEG and at least 24 h continuous EEG monitoring. All C19 patients had at least one SARS-CoV-2 positive test prior to the initiation of EEG or during EEG monitoring. Patients who tested positive for SARS-CoV-2 were admitted for symptomatic C19 disease, and not incidentally testing positive or testing positive from a previous infection. Patient’s mental status was assessed at bedside by registered EEG technologists at the start of EEG. They classify patients into awake, lethargy, stupor and coma states based on their level of responsiveness to standard stimulation and physical examination: ‘awake’ is able to confirm orientation to place, person, time, answer a general knowledge question and follow eye opening/closing on command; ‘lethargy’ is responsive to verbal questions/commands but is slow to respond and has inconsistent response; ‘Stupor’ is no response to verbal commands but responsive with eye opening and other movements on physical stimulation; ‘Coma’ is no eye opening in response to physical stimulation. A complete list of underlying conditions and comorbidity for every patient is shown in Table 4. Other exclusion criteria included acute or worsening chronic brain pathologies, hemorrhage or tumors and acute onset of systemic diseases.

**Table 4:** Summary of clinical comorbidity for each patient.

C19 (n = 31)		Ct (n = 38)	
id	Comorbidity	id	Comorbidity
1	Cancer, heart murmur	1	Transient ischemic attack, HLD, stenosis, carotid artery occlusion
2	EtOH, cirrhosis, acte kidney injury, varices	2	Dysphagia, nephrolithiasis
2	Rheumatoid arthritis	3	Cardiomyopathy
4	DM, psychiatric diagnosis	4	Multiple sclerosis
5	Cardiac arrest	5	DM, HPL, gout, ca, urinary tract infection
6	HLD	6	End stage renal disease, DM, subdural hematoma
7	Asthma, DM	7	N/A
8	N/A	8	DM
9	Dyslipidemia, obesity	9	Cirrhosis
10	Heart failure, atrial fibrillation, monoclonal gammopathy of undetermined significance (MGUS), pericardial effusion	10	Atrial fibrillation, HLD, cancer
11	COPD, headache, recurrent meningitis	11	Carotid stenosis
12	HLD	12	Melanoma, bening prostatic hyperplasia, spinal stenosis, cardiomyopathy
13	Atrial fibrillation, DM	13	Multiple sclerosis, depression, anxiety, hypothyroid
14	Prostate cancer	14	Chronic Lymphocytic Leukemia, DM, HPL, atrial fibrillation
15	DM, heart failure, deep vein thrombosis, hypothyroid, HLD	15	DM, HPL, hypothyroid, COPD, benign paroxysmal positional vertigo
16	COPD, congestive heart failure, hemicolectomy	16	Crohn’s, COPD, black lung disease, herpes simplex
17	HLD, glaucoma, AV disorder	17	Headache
18	Peripheral artery disease, osteomyelitis	18	Migraine, depression, postural orthostatic tachycardia syndrome
19	Liver failure	19	Parkinson disease
20	Autism, tuberous sclerosis	20	DM, hepatitis C
21	Cirrhosis, Crohn’s disease, vasculitis, hypothyroidism, DM, obstructive sleep apnea, obesity	21	HPL, hypothyroidism, paroxismal atrial fibrillation, childhood apraxia of speech, atrial valve replacement
22	DM	22	Peripheral vascular disease, heart transplant, HLD, DM, hypothyroidism
23	Schizoaffective, EtOH	23	HLD, COPD, giant cell arteritis
24	N/A	24	Parkinson disease, DM
25	N/A	25	Atrial fibrillation, subdural hematoma
26	N/A	26	Atrial fibrillation, HLD, GERD, complex migraine, vertigo, left common iliac art aneurysm
27	Alzheimer, bipolar, schizophrenia, hypothermia, DM, HLD	27	N/A
28	Cirrhosis, DM, Gerd	28	Dementia, depression, hypothoroidism, nephrolithiasis, congestive heart failure
29	HLD	29	Septic arthritis, DM, COPD, asthma, obesity, HLD
30	EtOH	30	HPL, DM, atrioventricular stenosis, atrial fibrillation
31	DM, aortic dissection	31	Cirrhosis, hepatopulmonary syndrome, liver transplant
		32	DM, dementia
		33	Cardiac arrest
		34	N/A
		35	DM, Alzheimer disease
		36	N/A
		37	N/A
		38	Depression

*DM* Diabetes mellitus, *HLD* Hypersensitivity lung disease, *HPL* Hyperlipidemia.

### EEG preprocessing

Pre-processing of EEG data, feature extraction, statistics, and machine learning were performed using MatLab (MathWorks, Natick, MA). EEGs were collected at a sampling rate of 200 Hz. A high-pass filter with a passband frequency of 1 Hz and a notch filter with a stop-band of 57.5–62.5 Hz were applied to all recordings. All EEGs were first visually inspected to confirm signal quality for each channel; channels considered to be of low or irretrievable quality were excluded from the study. Waveforms in each channel were divided into 1-s epochs, and each epoch was tested for the presence of artifacts using a previously validated method based on automated detection of artifacts by a SVM^34^. Briefly, this analytical pipeline previously achieved 85% accuracy when validated using external EEG datasets in variable disease conditions. Our team used similar computational principles to design highly accurate companion pipelines for artifact detection in awake, behaving rodent and canine subjects, which generate far more complex artifacts than resting state human EEG. Epochs not containing artifacts were included in further analysis; all other epochs were excluded. Rejection of channels was based on initial qualitative inspection of visually overt artifacts, followed by automated identification of artifacts and rejection of channels with less than 1.5 min of artifact-free epochs. Maximum EEG recording duration per subject was 15 min.

### Feature extraction

From the remaining artifact-free epochs of each recording, the following features were calculated for all channels: band-wise PSD Power Spectral Density (PSD) for all channels (average channel montage as in Fig. 1, or individual channel montage as in Fig. 2), and band-wise Phase-Amplitude Coupling (PAC).

To create the band-wise PSD, a periodogram was gathered from artifact-free 1-s epochs, and then these periodograms were averaged together for each channel within each subject using the: “periodogram” function in MATLAB which returns the two-sided periodogram, whereby:

[pxx,f] = periodogram(x,window,f,fs).

f: at least two elements, frequencies in cycles per unit time.

fs: sample rate or number of samples per unit time.

The band-wise PSD is then calculated by taking the average of all bins within each of the following five frequency bands: Delta (1–4 Hz), Theta (5–9 Hz), Alpha (10–13 Hz), Beta (14–32 Hz), and Low Gamma (33–52 Hz). This yielded 5 PSD features for every channel included. PAC was calculated using the Modulation Index (MI) method^37^. The center frequencies used for phase included all the even numbers from 2 to 20. The center frequencies used for amplitude included all multiples of 3 from 30 to 54. MI was measured for each pair of phase and amplitude frequencies (90 total pairs) for each channel, including only those time points for which there were 5 or more consecutive artifact-free eyes-open epochs. This yielded a 9 × 10 MI matrix, for every channel of every subject, with each row corresponding to one phase center frequency, and each column corresponding to one amplitude center frequency. This MI matrix was converted to band-wise PAC for the following 3 pairs of bands: Delta—Low gamma, Theta—Low Gamma, Alpha—Low Gamma, and Beta—Low Gamma. Other band-wise PAC were computed for the following 4 pairs of bands: Delta—Medium Gamma, Theta—Medium Gamma, Alpha—Medium Gamma, and Beta—Medium Gamma. This conversion was accomplished by averaging across the appropriate regions of the MI matrix. This yielded 4 PAC features for each channel, and a maximum of 64 PAC features per subject (4 band-pairs × 16 channels).

### Statistical analyses

We used paired two-tailed t-tests to compare the band-wise PSDs between the PCR positive and PCR native age-matched groups^38^. Our PSD data meet assumptions for conducting parametric tests, including homogeneity of variance, and normally distributed data. This was assessed via histograms, QQ plots, and Shapiro–Wilk test. Additionally, means are similar to medians for all groups. We used two-tailed Wilcoxon rank-sum tests to compare the band-wise PAC from the two groups for each of 4 band pairs and 16 channels (64 tests in total). We chose to use non-parametric statistical test for PAC because values are constrained between 0 and 1, and are therefore less likely to follow a normal distribution, as required by Student's t-test. Statistical significance was established throughout at *p* < 0.05. Statistical analysis of average channel EEG power in multiple frequency bands was adjusted according to Boneferroni correction for the number of bands (delta, theta, alpha, beta, gamma, i.e. p values multiplied by 5), and for the number of channels multiplied by the number of individual channels (i.e. 5 bands × 12 channels = 60).

### Classification and prediction

The features described above (band-wise PSD) were used to create a feature-set for training binary classification algorithms. In particular, only mean power values that were shown to be significantly different between groups were selected for algorithm training (for example mean delta, theta, alpha and beta for younger subjects, and theta, beta and gamma for older subjects in Fig. 1). We validated the classifiers using k-folds cross validation using *k* = 4. Classification accuracy was calculated within the k-folds cross validation by counting the number of out-of-sample predicted labels that matched the true label of the sample, and dividing this total by the number of samples (n = 24 for age below 70 group and n = 36 for age above 70 group).

## Supplementary Information

Supplementary Information.

## Data Availability

The datasets generated during and/or analyzed during the current study are available from the corresponding author on reasonable request.
