# Supplementary material for: SARS-CoV-2 alters neural synchronies in the brain with more severe effects in younger individuals
Source: Sci Rep. 2023 Feb 20;13:2942. doi: 10.1038/s41598-023-29856-7 (PMC9940054; doi:10.1038/s41598-023-29856-7)
Supplement: Supplementary file 1 — Supplementary Information. [file 41598_2023_29856_MOESM1_ESM.docx]

**Supplemental Information File**

*Supplemental Figures 1-6)*


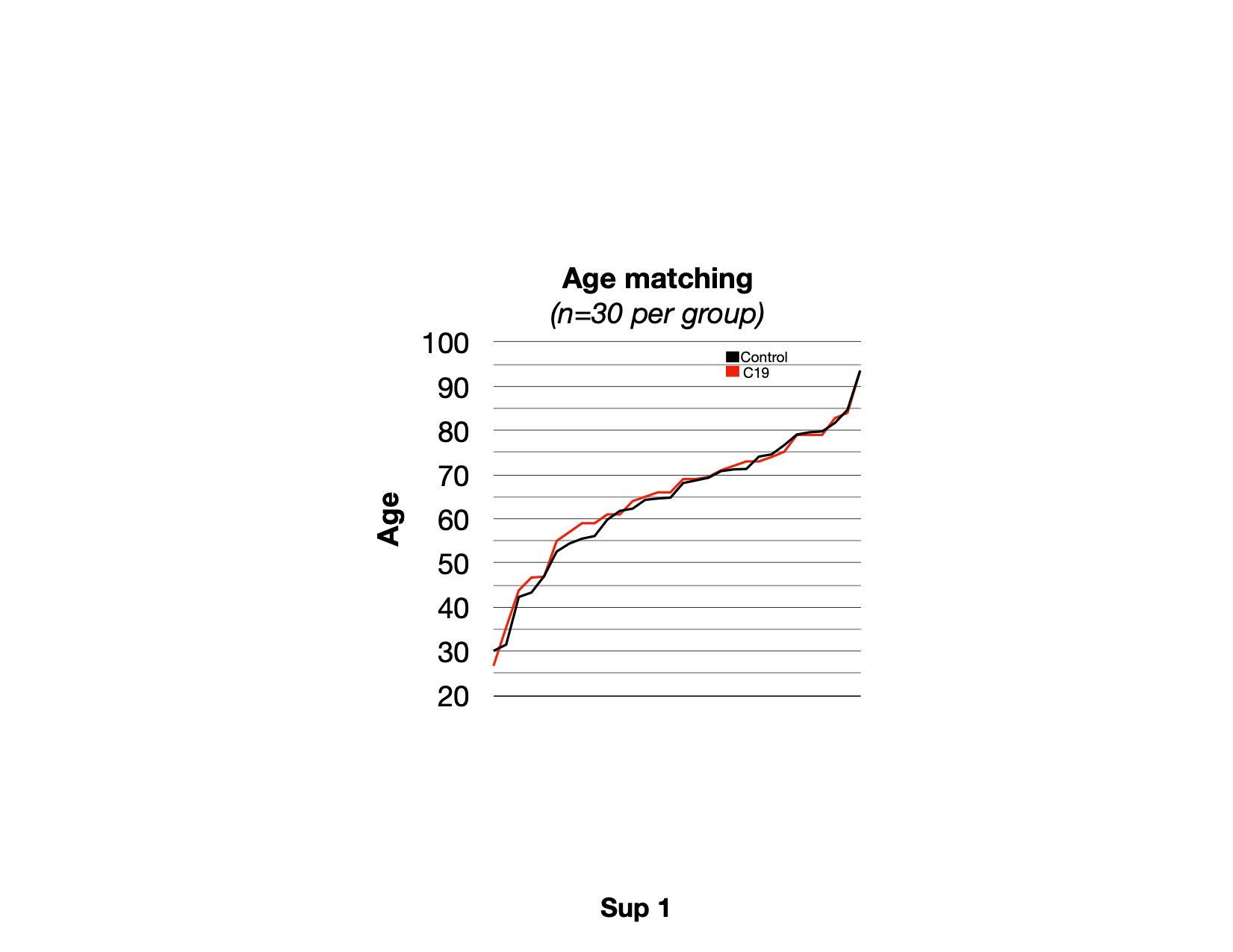


Supplemental Figure 1 (Age Matching): Age distribution for subject pairs with a matching requirement of maximum 5 years difference in C19 (red) and control groups (black; n=30 subjects per group).
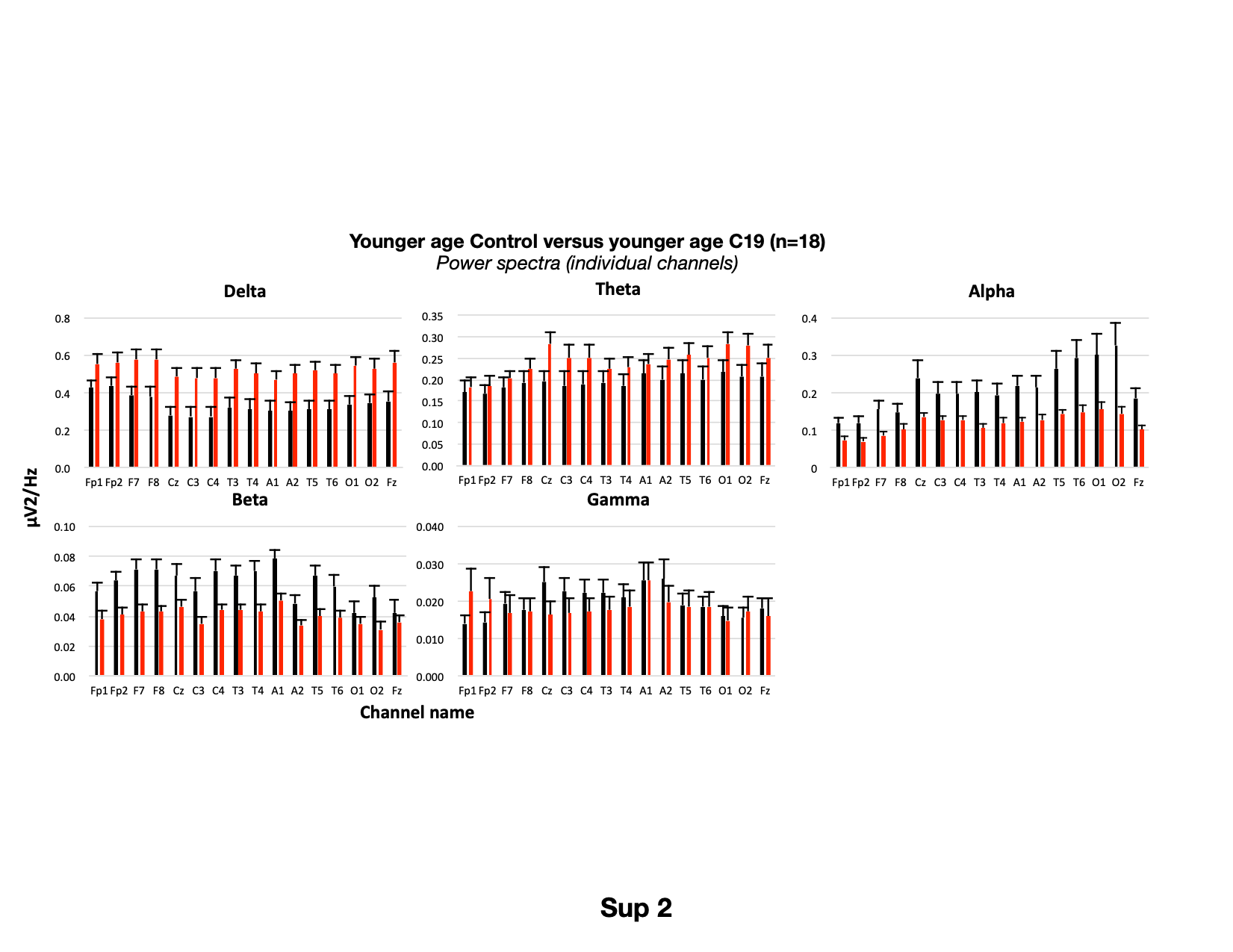


Supplemental Figure 2 (PSD Young Age Control vs Young Age C-19): Power spectral density in 16 individual EEG channels in age below 70 (younger) control and C19 subjects (n=18 per group) in the frequency bands delta, theta, alpha, beta and low gamma. No statistically significant difference was noted between groups in any individual channel.

Supplemental Figure 3 (PAC Control vs C-19): Phase-amplitude coupling (PAC) in 16 individual EEG channels in age-matched control and C19 subjects (n=30 per group) between medium gamma and delta, theta, alpha, beta respectively (upper row), as well as between low gamma and delta, theta, alpha, beta respectively (lower row). No statically significant difference was noted between groups in any individual channel.


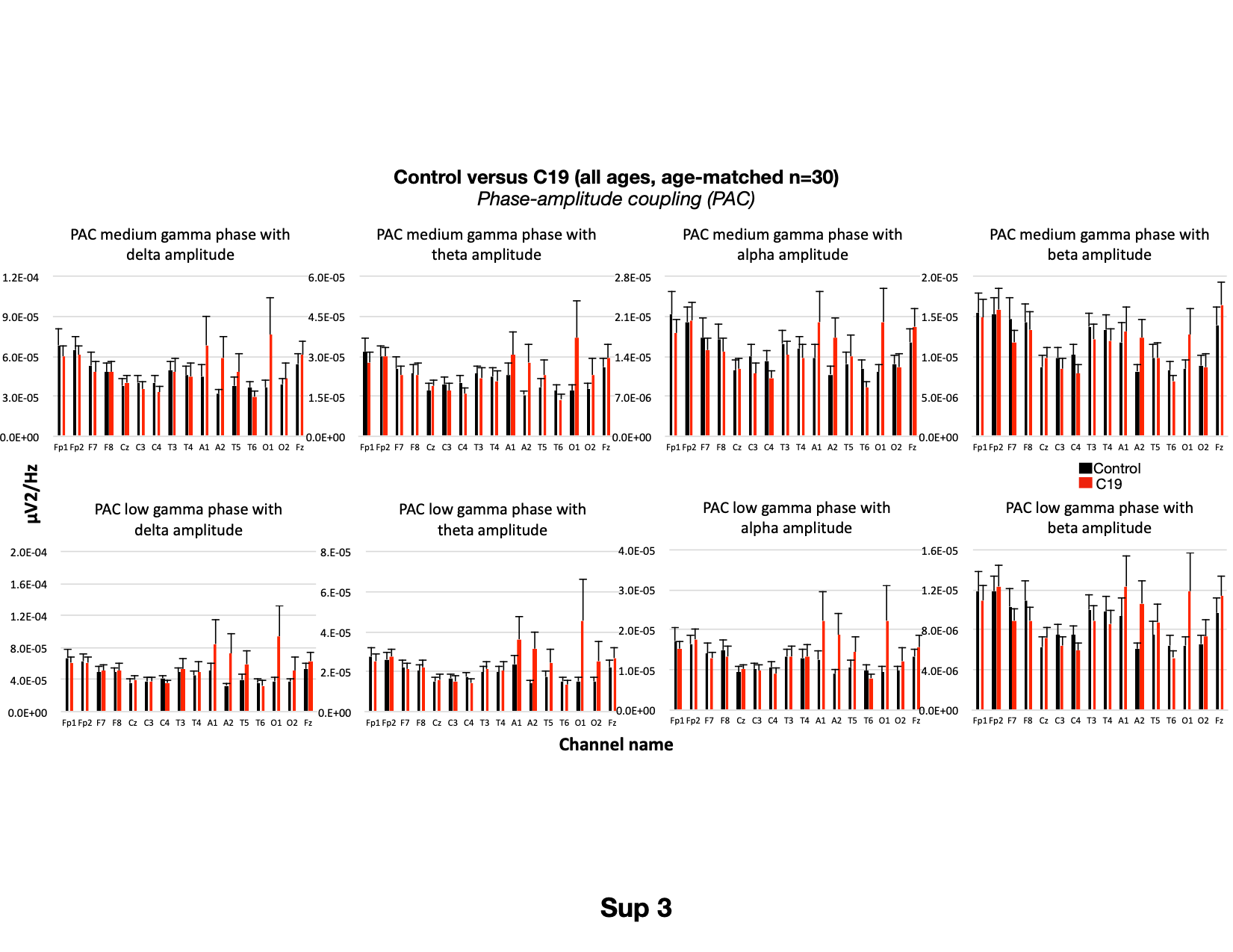


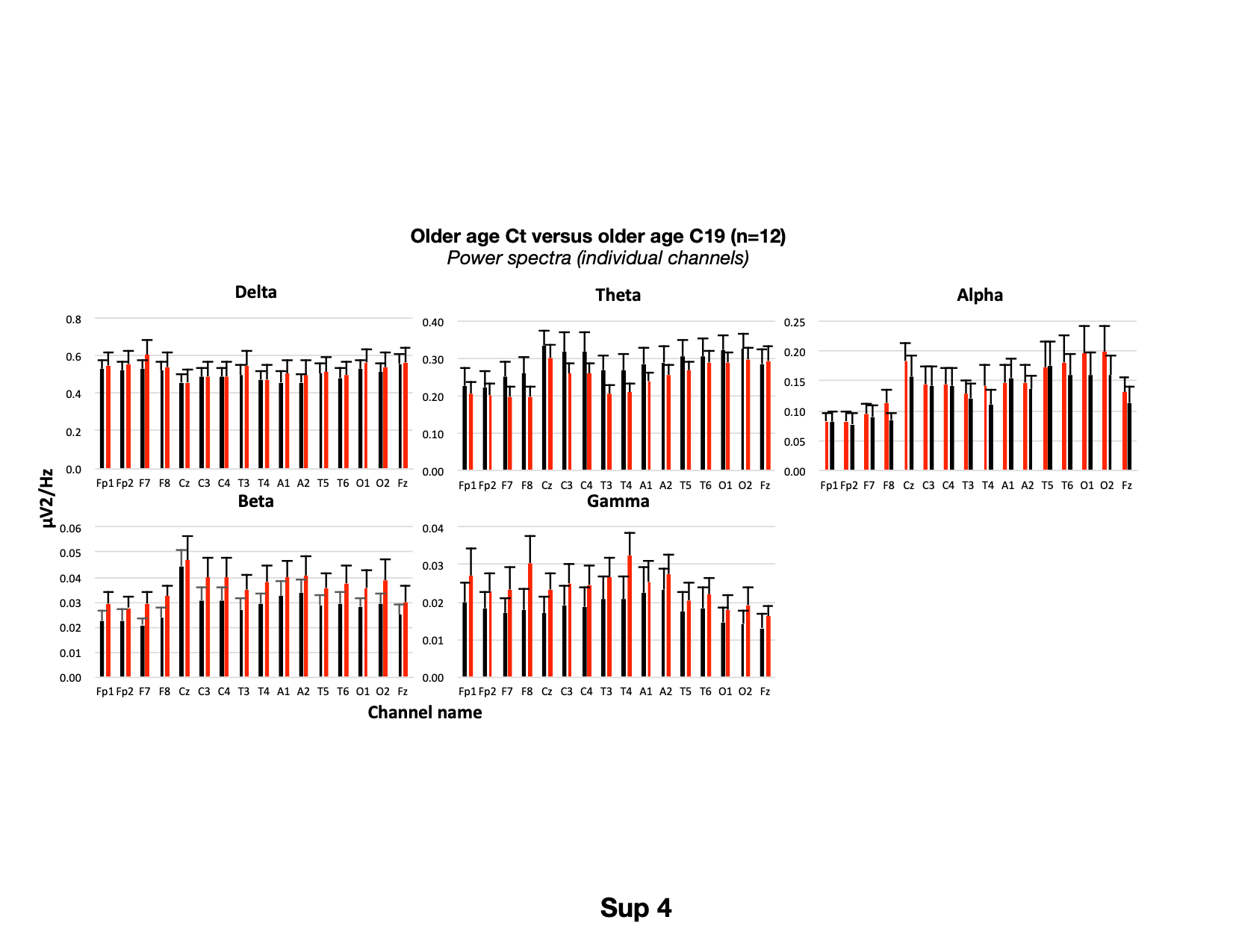


Supplemental Figure 4 (PSD Old Age Control Vs Old Age C-19): Power spectral density in 16 individual EEG channels in age above 70 (older) control and C19 subjects (n=12 per group) in the frequency bands delta, theta, alpha, beta and low gamma. No significant difference was noted.

Supplemental Figure 5 (PSD Old Age C-19 vs Young Age C-19): Power spectral density in 16
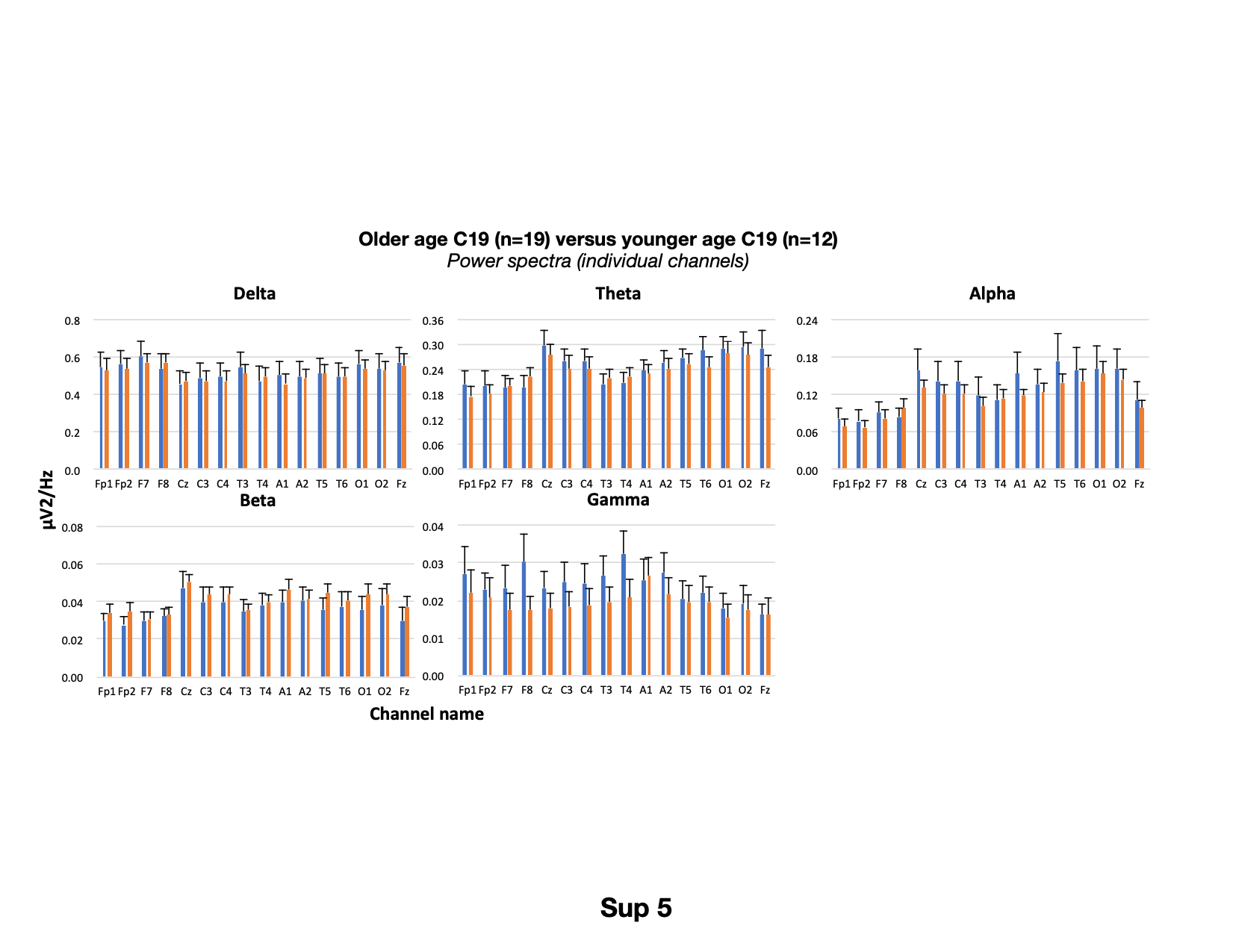
 individual EEG channels in age above 70 (older) C19 subjects (n=19) and age below 70 (younger) C19 subjects (n=12) in the frequency bands delta, theta, alpha, beta and low gamma. No significant difference was noted.

Analysis of the following clinical variables mitigates the concern for potential confounds:

(1)     Reason for EEG (Altered mental status, Loss of Consciousness, Motor Weakness or Ruling Out a Seizure)

(2)       Cerebrovascular accidents

(3)     Epilepsy

(4)     Hypertension

(5)     Prior episode of hypotension during hospitalization

(6)    Prior need for ventilation during hospitalization

(7)     Hypotension on EEG day

(8)     Imaging Results (Hemorrhage , no acute lesion, infarct or tumor)

(9)     Disposition type (Home, acute rehabilitation facility, subacute rehabilitation facility, skilled nursing facility, or expired)

(10) Creatinine (in mg/dl) (11) Total bilirubin (in mg/dl)

Statistical analysis of the 11 potentially confounding variables above between the two groups using exact logistic regression to predict C19 status from each parameter separately (with linear age added to each model because subjects were age-matched) revealed no significant difference (Table 1).

Table 1. Tests of interaction between predictor & age were conducted; Hosmer-Lemeshow tests of goodness of fit were applied. Bonferroni-corrected p-values are reported here for the mid-P score test.
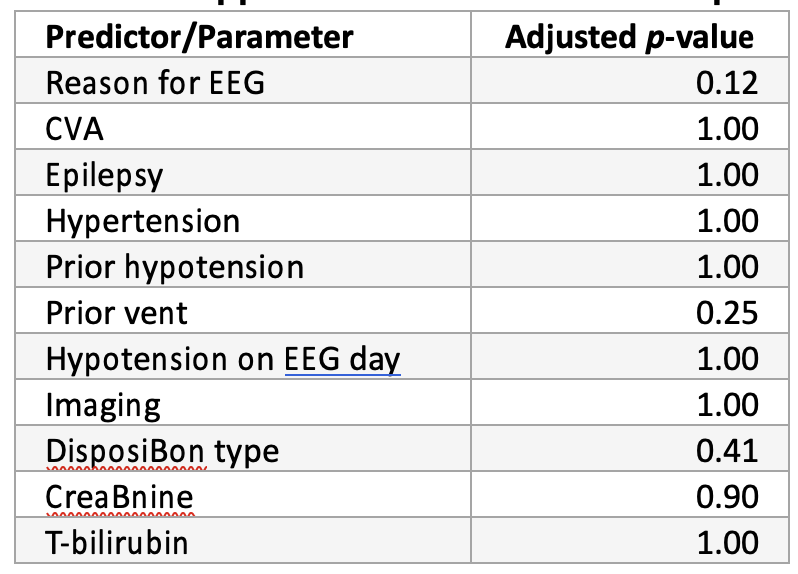


Four of the above parameters were further subcategorized in Table 2 for an accurate identification of confounders between the two groups.

Table 2. Subcategories of Parameters
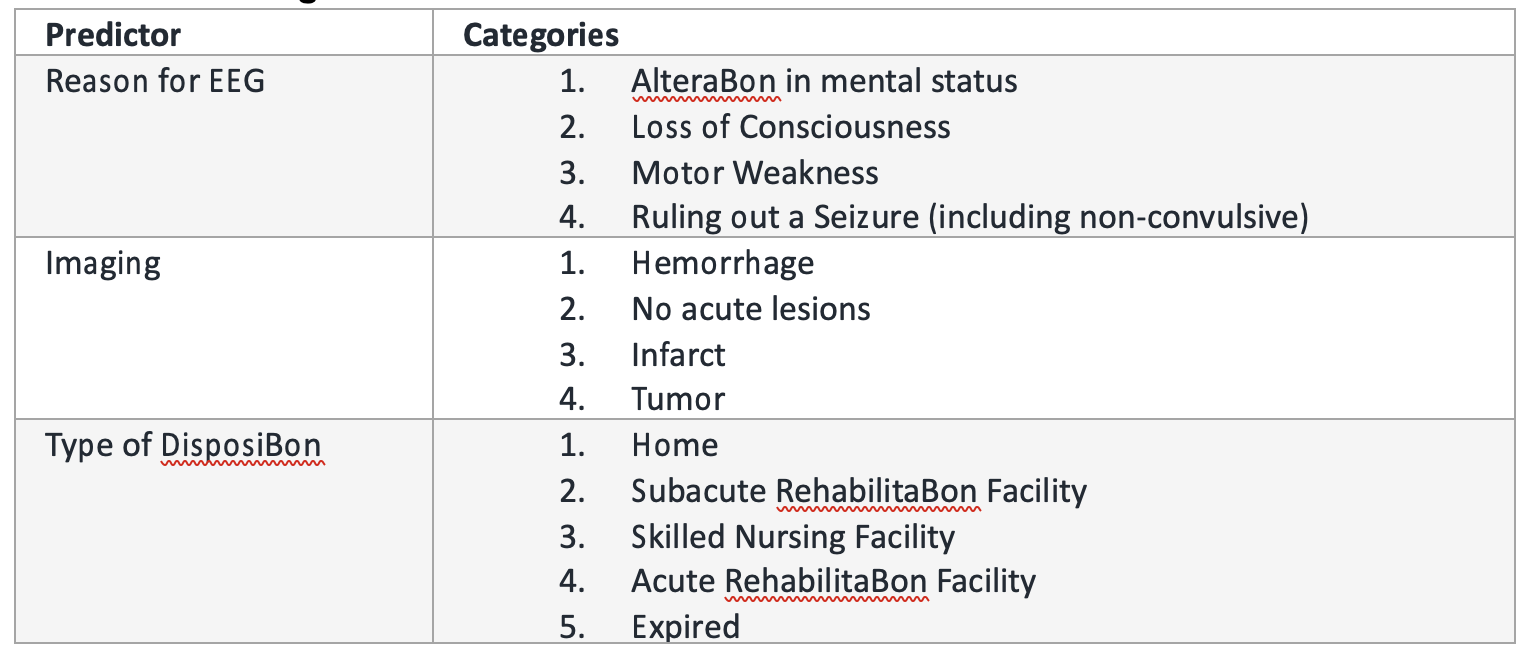


The above statistical methodology was based on clinical data from EPIC medical records for every patient in our study. None of the above parameters including renal status, nor other co-morbid medical conditions, were predictive of the C19 status. Thus, the quantitative EEG findings specific to the C19 group are less likely to be attributed to co-morbid illnesses.

Supplemental Figure 6 (Clinical Confounders): Tests of interaction between clinical conditions as predictors and age. Hosmer-Lemeshow tests of goodness of fit were applied and Bonferroni-corrected p-values are reported for the mid-P score test.
